# Supplementary material for: Are cardiovascular health measures heritable across three generations of families in Soweto, South Africa? A cross-sectional analysis using the random family method
Source: BMJ Open. 2022 Sep 23;12(9):e059910. doi: 10.1136/bmjopen-2021-059910 (PMC9511591; doi:10.1136/bmjopen-2021-059910)
Supplement: Supplementary data [file bmjopen-2021-059910supp001.pdf]

Appendix: *Additional information on cardiovascular assessment methods*

Arterial stiffness (carotid-femoral pulse wave velocity - PWV) was estimated, with tonometry of the carotid artery during inflation of an appropriate size femoral cuff. Pulse wave analysis (for central pressure estimation) and PWV measurement were set at 10 second intervals. Duplicate measures of PWV were taken and if the difference between PWV measures was ≥0.5 m/s, a third measure was taken and the average of two readings within 0.5m/s of each other used for analysis. All measures were taken on the right side with the participant resting supine for 10 minutes prior to measurement, and using the direct distance method to estimate aortic path length[1]. A total of 4 trained operators performed the PWV measurements after confirming inter-observer variability was acceptable (< 0.5 m/s).

Left ventricular mass (LVM) was measured in 2D mode with transthoracic echocardiography following the American Society of Echocardiography (ASC) protocol[2]. The 2D mode has been shown to be superior to M-Mode for studies of LVM within families[3]. LV mass was assessed at end-diastole perpendicular to the long axis of the left ventricle. The Devereux formula was used to calculate LVM: LVM (g) = 0.8 x 1.04 ((LVDd + IVSd + LVPWd)3 – LVDd3) + 0.6 where LVDd=left ventricular diastolic diameter; IVSd= intraventricular septal diameter, LVPWd= left ventricular posterior wall thickness in diastole[4].

Carotid intima-media thickness (cIMT) was determined using high resolution B-mode ultrasound employing a linear array 7.5 MHz probe as recommended[5]. Images of at least 1 cm length were obtained of the far wall of the distal portion of the left common carotid artery (CCA) from an optimal angle of incidence (defined as the longitudinal angle of approach where both branches of the internal and external carotid artery are visualised simultaneously). Semi-automated border detection and quality control software were used to calculate cIMT, with at least 3 measurements obtained from the left side and the mean used for analysis. Previous studies have reported no major differences between left and right CCA IMT in associations with cardiovascular disease[6]. All ultrasound measures were taken with the Mindray DC-70 Ultrasound system (Mindray, Shenzen China).

1. Van Bortel LM, Laurent S, Boutouyrie P, et al. Expert consensus document on the measurement of aortic stiffness in daily practice using carotid-femoral pulse wave velocity. *Journal of hypertension* 2012;30(3):445-48.

2. Lang RM, Badano LP, Mor-Avi V, et al. Recommendations for cardiac chamber quantification by echocardiography in adults: an update from the American Society of Echocardiography and the European Association of Cardiovascular Imaging. *European Heart Journal-Cardiovascular Imaging* 2015;16(3):233-71.
3. Shah SJ, Ober C, Lang RM. Two-Dimensional Echocardiography Is Superior to M-mode for the Determination of Left Ventricular Mass: Evidence from a Heritability Study. *Journal of Cardiac Failure* 2007;13(6):S155.
4. Devereux RB, Alonso DR, Lutas EM, et al. Echocardiographic assessment of left ventricular hypertrophy: comparison to necropsy findings. *The American journal of cardiology* 1986;57(6):450-58.
5. Touboul P-J, Hennerici M, Meairs S, et al. Mannheim carotid intima-media thickness and plaque consensus (2004–2006–2011). *Cerebrovascular diseases* 2012;34(4):290-96.
6. Bots ML, de Jong PT, Hofman A, et al. Left, right, near or far wall common carotid intima-media thickness measurements: associations with cardiovascular disease and lower extremity arterial atherosclerosis. *Journal of clinical epidemiology* 1997;50(7):801-07.
