# Supplementary material for: Are cardiovascular health measures heritable across three generations of families in Soweto, South Africa? A cross-sectional analysis using the random family method
Source: BMJ Open. 2022 Sep 23;12(9):e059910. doi: 10.1136/bmjopen-2021-059910 (PMC9511591; doi:10.1136/bmjopen-2021-059910)

Supplementary figure 1 showing the relationships between the heritability parameters for LVMI (adjusted for body surface areas) and carotid IMT (cIMT).

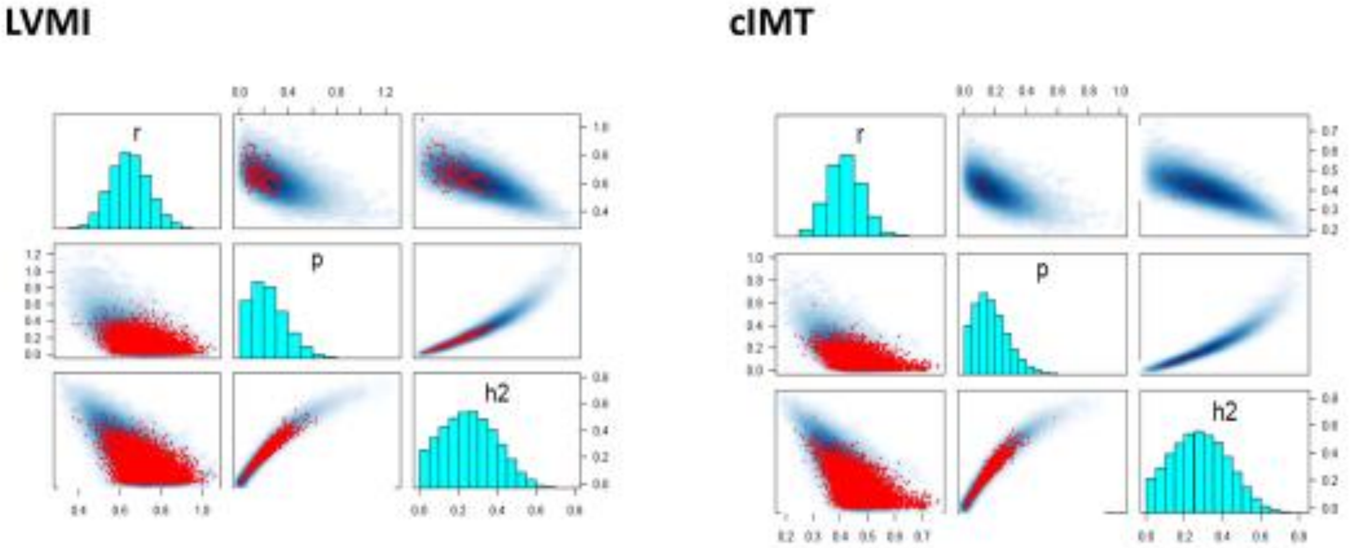

Supplement: Supplementary data [file bmjopen-2021-059910supp002.pdf]
